# Address

**Published:** 1876-04

**Authors:** James Taylor

**Affiliations:** President of the Board of Trustees


### DENTAL REGISTER.

Vol. XXX.] APRIL, 1876. [No. 4.

### ADDRESS.

BY DR. JAMES TAYLOR, PRESIDENT OF THE BOARD OF TRUSTEES.

*Delivered before the Graduating Class in the Ohio Dental College, March 2d, 1876.*

Gentlemen graduates:—You have just been formally inducted into the profession of your choice. We are gratified to know that your examinations have entitled you to this honor. We are assured that the session just closed has been one of hard labor and study, and that all have manifested an earnest desire to thoroughly qualify yourselves for the duties you are about to assume.

It has been rather the custom for the President of the Board of Trustees, after having conferred degrees, to give the class some sage counsel and prudent advice; how you should act, what you should do and what you should not do. We propose, however, on this occasion, to detain you a short time in telling you what has been done. This is called the centennial year, let us look then at what has been done for our profession in the last century. Feeling, however, that the time allotted me would not suffice to take a general review of the profession's progress, we shall confine ourself to the one great and paramount operation—that of filling teeth, and that we may not tax your patience too long, we shall confine our remarks almost exclusively to the preparation of the cavity, the instruments to be used, the different preparations of gold, how introduced, the improved methods of keeping the cavity dry, etc., etc.

Time will not permit the treatment of exposed pulps or nerves, etc., etc. But our subject is a review of the operation of filling teeth for the last hundred years. We shall not however, attempt to trace out in detail, the progress made the first fifty years of this period. The few works published during this time and the utter want of practical detail, gives but little to predicate much upon. We will therefore merely glance at a few facts from the first half of the century, to furnish a starting point for the further elucidation of the subject.

Hunter, who may be regarded as the first and really the greatest author of his day, and who wrote about the beginning of the century, gives no special directions how to prepare and fill the cavity. He does, however, say, "that when the cavity becomes too large to keep in any of the substances named, that a hole may be drilled through the strong part of the tooth, after the tooth has been stopped and a peg may be put into the hole so as to keep in the lead, gold, etc."

This appears to have been the first idea of retaining points' the thought, like many others, lay slumbering near a century before revived and put into practical operation. Fox's directions are, remove extraneous matter, dry the cavity, then introduce a piece of gold or tin foil leaf. His instruments consisted of a hook for removing extraneous matter, a straight and curved instrument for pushing the stopping into the tooth and a burnisher for polishing.

Desirabode says a good deal about the different materials used. Darcet's metal, succedanium, gold, platinum, lead, etc., and directs that the gums and lips should be covered with a layer of cotton. This is about the first direction looking to this object, and he gives directions how to prepare the foil. He says, "Tear off a piece and roll it between the fingers, a little larger than the cavity to be filled, place the finger of the left hand at the opening of the cavity, while with a small sized blunt pointed plugger he forces in the metal." He formally disapproves of the practice of leaving the plug above the edge of the teeth, to be pressed down by the act of mastication, thinking that the dentist should perform the operation himself. Lefoulon, while he does not classify and describe the cavities to be filled, does speak of the manner of preparing gold, platinum and tin leaves. He says, "we form them into a species of tubes or little balls; or what is yet better, into several folds, leaving them of considerable length, then with a plugging instrument we introduce the metal gradually into the cavity, taking care so to pack it that it may bear equally upon all points." This is some progress and for a long time held sway as the mode of operating and indeed to this day many employ the same method. It is well to remark that during the early part of the century and particularly in France. Sensitive dentine was treated with the actual cautery.

Some of the early dentists speak of grooving the cavity, making it larger within than without or at the orifice, others contend that such cavities can not be well filled, preferring the cavity the same size all the way.

It is worthy of remark that all the best operators of that day as well as now; speak of gold as the best material to be used, condemning as we do amalgam, Darcet's metal, etc.

Perhaps no man during this period stood higher and deservedly so, than Dr. Koecker. He spent several years in this country and particularly in Philadelphia, where he was located in 1811. His general views with reference to the preparation of the mouth for this operation are worthy of all commendation and should be more carefully observed at this time, and while his special directions how to prepare and fill a difficult cavity are very meager indeed, yet that his operations were thorough and perfect for his day, no one can deny. In speaking of certain injurious methods of filling teeth. He says, "I recollect some practices of the profession which can not be too strongly reprobated and among others, that of using a common drill, turned by a bow and string, for making the cavity of the tooth sufficiently large and otherwise adopted for the reception and retention of the plug, without considering the almost utter impossibility of giving that direction to the drill which the caries has taken, with any degree of certainty and the frequent destruction of important parts of the bony structure as well as of the lining membrane, the excessive irritation and pain produced by the rapid friction of such instrument is alone sufficient to destroy the vitality of the teeth."

This drill is slow, gentlemen, compared to our present engine. But he remarks "what is more surprising and repugnant is after the tooth is thus prepared for the reception of the stopping; some operators actually employ a hammer and punch to drive the metal into the cavity of the tooth.* He does not say, who had the honor of introducing the hammer or to use a milder term, the mallet, into dental practice, but he speaks of the fact and it is well to look at the first dawning thought which inaugurates a new method. In connection with this it is well enough to state the fact that in 1843 or 4, Dr. Madary, then of this city, very generally used a mallet for hammering in the gold, and made quite a stir in the matter. Dr. Koecker speaks of some alarming consequences proceeding from this barbarous practice.

We might say that up to 1830 or even 40, we find no special classification of cavities, no general and definite description of the instruments for preparing the cavities and none whatever for the shape and character of those for introducing the gold. Up to this time many beginners attempted to

*See Koecker's work, page 209.

fill the proximate cavities by stopping the entire space between the teeth. We have seen teeth filled in this way by men claiming great skill. We think it fair to say, that up to this time, that is 1840, students could not get as much real practical knowledge by carefully reading and studying all the works then published on dental surgery as they can now acquire in one week in any of our well regulated dental colleges.

We do not mean by this that our dental literature was a failure. Far from it, we had many excellent works on diseases of the teeth and mouth, physiological and pathological, but very little that was practical.

We can claim a Hunter, a Fox, a Bell, Desirabode, Fitch Delabarre, Lefoulon, Blake, Nasmyth, Koecker and many others; whose works show great research and have done much to place the profession on the solid basis it now occupies. Indeed all these authors take dentistry as a part and parcel of general medicine and surgery, and, to this fact, as much as any other, we are indebted for our medical standing to-day.

You must recollect gentlemen, that mere mechanical manipulation never can do much to elevate the profession. It takes true scientific knowledge to guide this aright.

We may safely say that there is no operation in surgery that requires more skill, more true scientific knowledge than the operation under review. None which demands more of all this for its successful accomplishment. Study then these authors, for it is through these sources we may trace out the gradual development of our science.

We have now taken a hasty review of this operation up to 1839 and if we live until June 1st, 1889 we propose to celebrate the semi-centennial of dental surgery in the United States. For June 1st, 1839 forms an epoch in our profession, which has given an impetus to dental progress, perhaps unprecedented in any other profession. It was then the profession threw off the yoke of bondage and struck for a universal brotherhood. It was then the old mantle of distrust and secrecy was thrown off. It was then the declaration of dental independence was proclaimed and the first number of the American Journal of Dental Science was published.

Light begins now to break in and we shall be able only to glance at a few of the leading events which mark our progress in even this one operation.

Permit me, however, to digress one moment, to draw attention to an article in the fifth number of the first volume of the journal to which we have alluded.

It is a list of 175 subscribers to the journal, but this would not begin to sustain such a publication, and these few subscribers took 488 copies of the work. Dr. C. A. Harris 40, Dr. Eliazer Parmley 40, and eleven gentlemen 20 each. We have always regretted our name is not on this list, although we took two copies of the first volume, one being sent to Dr. Taliaferro—whose name is on the list and from an oversight, ours left out. But gentlemen look over sometimes this list of names and see what a noble brotherhood they were. Only a few, alas! how few, now survive to rejoice over the result of their labors.

This number of the journal also contains the first article on filling teeth; styled "My method of filling the front teeth," by S. L. Stringfellow. A short, concise and best description of the operation up to that time.

Strange that this operation, although considered so important, and with that of filing the only remedial agency for their preservation, should have received in its special method so little practical instruction. For even during the first five years of the publication of the journal, but little was brought forth except what might be regarded as of a general character. Being the great operation, the one which should stand out, and now does, above all others in importance, we would have supposed, that, the most minute and careful description would have been given with reference to the best method of filling every condition of decay.

True, we have something about the difficulty of filling when the decay is between the teeth, but not how to overcome that difficulty. We have something and far more about sensitive dentine, and how to cap and protect the nerve.

Yet but little how to prepare the cavity, keep it dry and put in the gold. Dr. C. A. Harris is the first author who gives anything like explicit directions how to prepare a cavity. Stand when operating, hold the instrument, keep the teeth dry and pack the gold. He gives a somewhat careful account of the location of the different cavities, naming them according to their locality, etc.

This work forms an epoch indeed in the right direction. He takes the operation as he finds it and as performed by the most successful and best operators, and very carefully and accurately describes it, so that any intelligent student may understand it. Compared with previous publications on this subject, he stands out in bold relief. It is easily seen he was a careful and fine manipulator, and describes what he was in the habit of doing.

His method of preparing the cavity with different shaped excavators and the common bur head drill was perfect in its way, and his manner of preparing the gold and introducing it is in accordance with the then most prevalent method, commencing with a strip of gold foil at the bottom of the cavity, folding and packing into every part of the cavity until it is full. Our best operators arrived at results which have made the profession what it is to-day. True we make successful operations now with our present appliances, which were then not attainable.

Serrated plugging instruments were not then thought of, but condensers were. They were used as early as 1832. We know not who introduced the sharp pointed instruments for packing the gold into the cavity or the serrated instruments for condensing the gold, yet both were in use as early as 1830 or 32. Before the days of dental periodicals we knew but little of each other's mode of operating or the kind of instruments used by each other. The profession was rather exclusive and kept instrument cases with keys to them.

In vol. iv. DENTAL REGISTER, 1850, we published a series of articles on filling teeth, giving much the same classification of cavities as those named by Dr. Harris, with some other combinations, presenting as then an entirely new method of preparing and inserting the gold, also describing the particular shape of each class of cavities for the better retention of the plug.

This was the introduction of the block or cylinder mode of filling, the main object of this was the introducing of the gold in a perfect laminar condition, avoiding as much as possible the crumbling of the foil and securing each layer of gold in a lamellated state from the bottom of the cavity to the outer surface of the plug, each cylinder acting as a wedge driven into the cavity of the tooth. In this arrangement of the gold, the ends of the cylinders form the exposed or outer portion of the plug, and each lamina of gold passing into the bottom of the cavity, we have a condition of gold and particularly in the form of foil to withstand in a perfect manner the friction of the tooth pick and mastication. This method requires more care and labor in the preparation of the cavity and the gold, but this is more than compensated by the rapidity with which the gold can be introduced; before the introduction of the rubber dam this was an advantage of far geater importance than at present, still there are many cases now and always will be, where the most expeditious method of introducing the gold is of immense value. In looking over recently the description we then gave of this operation, we see but little more to be said, than you will find in the articles to which we alluded.

The method necessitated the use of plugging plyers or forceps, for handling the gold and carrying it to its place for consolidation.

Appliances since introduced, facilitate this method as much as any other, while they have secured for building up teeth and some conditions of contour fillings, a more practical and better method. But in all approximate cavities where the right form can be secured, with soft foil for all or nearly all of the filling, we secure results which have proved more satisfactory than any other. In central cavities on the grinding surface of the teeth, hard adhesive foil can be used with equal facility and as fine results as by any other method. The mallet comes in play here as well as elsewhere, but from the perfect arrangement of the gold much less malleting force is necessary.

In vol. viii. DENTAL REGISTER, page 198, we have an excellent article from Dr. A. S. Talbert, of Lexington, Ky., on the preparation of cavities, and as he gives about the first special directions with reference to retaining points, we will give his remarks on that point in full. After describing the method of making the drills for the purpose, he remarks:

"That with these exceedingly hard and sharp drills you have only to make a few rotations; at any point or points in the cavity that may suit your convenience or that the condition of decay may demand; care being taken to hold a steady hand, especially with the larger drills; which, however, need not be larger than a knitting needle or one-sixteenth part of an inch in diameter; while the smaller ones may be one-fourth as large or even less. The operator unaccustomed to the use of these instruments, will be astonished at the rapidity with which he is now able to prepare points in the cavity, which shall serve as fastenings to his plug. He is also better enabled to make choice where these points shall be cut. Selecting them remote from the pulp and at such places as would be impracticable to use an ordinary excavator."

You see these retaining points were accurately described and recommended over twenty years ago.

About this time, that is twenty years since, much discussion was had and experiments made with reference to different preparations of gold and the manner of using them. Sponge gold and crystal gold were being introduced. Adhesive gold foil also was attracting much attention. The idea of welding the different particles of gold in the filling, into one solid mass, soon became the almost controlling thought of the profession. That preparation of gold which best secured this object, was the perfection for filling teeth. It is possible that other qualities equally important may have been overlooked.

In vol. vii. DENTAL REGISTER, page 245, is an article from the pen of Dr. Dwinell, of New York, on sponge gold, crystal gold and foil; recommending highly, sponge gold as possessing "in an eminent degree, all the desirable qualities of the above, viz., toughness, compactness, pliability, together with plasticity with the highest degree of adhesiveness."

The merits and particular properties of these forms of gold, were pretty fully brought out at that time by Drs. Dwinell and J. D. White, with a review of the same by J. T. not, however, your speaker.

In vol. xi. DENTAL REGISTER, page 371, we find the following method of filling teeth with dead nerves to avoid abscess. The author says: "After having perfectly excavated the cavity and shaped it to your mind, and rimmed out the fang as far up as possible, I take a piece of wire of any tough metal, and after having drawn it to a firm point and burnished smooth, dip it into creosote and force it to the apex of the fang if possible. Fill the fang around the wire close and solid, being careful to keep it in a direct line. Proceed to fill and finish the main cavity, then with a pair of flat nosed plyers, withdraw the wire, thus leaving a small tube. All that is necessary for teeth having more than one fang, is to add a wire for each fang. This practice was recommended as late as 1858 and the author, W. H. Atkinson, claims to have pursued it for twenty years. We allude to this to show how very slow advance is sometimes made, not that we would advise you to hold on to a bad practice twenty years.

In vol. x. DENTAL REGISTER, page 232, (1856) we have an article from the pen of Dr. Arthur, on an improved method of using gold foil, which is worthy of special notice in this connection, and particularly so as it has developed into what may now be regarded as the most popular mode of practice. He remarks, "There are two methods in which gold may be prepared. It may either be used in the form of pieces cut from a rope, or the sheet may be cut up or torn into small pieces, without folding or rolling it up." He further remarks that, "In preparing the pellets, the sheet of No. 6, gold foil should be cut into strips according to the size of the cavity." "I generally, he further says, make my sheets into three strips."

"These sheets should be rolled up loosely, as loose as possible, laid upon a thin piece of platinum or other thin metallic plate, and heated over the spirit lamp. The degree of heat is unimportant if continued long enough, but it will be sufficient if the platinum is allowed, directly under the rope of gold, to reach a dull red heat, the roll is then cut up into pieces of a suitable size. The direction to roll up the strip loosely is important, as many persons who have attempted to use gold in this way, have failed because they have rolled it up tightly." "The instruments, "he remarks," used for this gold should terminate in two or more sharp points; the most convenient for general use, are those made with two, three or four points." The foil for this mode of operating must be adhesive.* He goes on further however to say, "After the first pieces are fixed, pellet after pellet is taken up with the point of the instrument and carried to the desired place, fixed by pressure against the gold previously put into the cavity and condensed, first with a large and then with smaller instruments, taking care to carry it against the sides of the cavity, thus going on, adding piece after piece until the cavity is filled."

The great difficulty, he admits, is to keep the first pellets to their place, this he does by holding them there, or recommends a plan suggested, he says, by "Dr. Louis Jack," which is, "Drill two small holes about the twelfth of an inch deep in a part of the cavity, where there is no danger of reaching the pulp, begin the operation at this point as there is no difficulty in getting the gol.l to remain in such small cavities and build the filling from these two points."

We have given the doctor's description quite fully, because you see that this method; to a great extent revolutionizes the whole operation of filling teeth. The cavities must or as a general thing should have retaining points. The foil must be adhesive in character, or any preparation of gold which may be used, and must be welded to and from these retaining points. The introduction of retaining points for the bet-

*It is but justice to say that Dr. James Leslie, of this city, was the first to introduce adhesive gold.

ter securing of fillings in the teeth, had been recommended by Dr. Talbert two years before this. The introduction of this method of filling teeth made it necessary that two other conditions should follow, viz: The gold, in whatever state it might be, as foil, crystals or sponge, should be adhesive, or perhaps more properly, cohesive, and that the instruments used for its introduction and consolidation, should be serrated so as to make deep sharp retaining pits in each layer of gold, as introduced. This hypothesis, we however believe unphilosophical and fallacious. If we mean by the adhesive or cohesive property of gold the quality of being welded, why make the filling full of small minute pits all of which can never be completely obliterated. But we turn attention to what has been said and done with these serrated pluggers. In vol. x. page 495, we have an article on the use of these instruments for "annealed gold." After alluding to the difficulties of many who had attempted to fill teeth with annealed foil by this method, he remarks, "In order to secure the perfect ad hesion of each layer of gold with the least difficulty, a surface should be produced and kept up, covered with pits and points, both of which should be acute. To secure this, it is necessary that the points on the ends of the pluggers, whether they have a single one or are composed of a dozen, should be absolutely sharp, and the depressions between the points likewise have the same acuteness. If either point or pit, or both, are blunt, a corresponding surface will be given to the gold, which will not favor near so well the adhesion of the pieces, as when sharper points are used. A pellet of annealed gold may, indeed, be easily made to adhere inseparably to a clear, smooth, unpolished surface of the same material, but it requires to be held in place at one point while another point is being condensed." "In a tooth, he remarks, this would be a troublesome matter. The serration of the points is intended to overcome this difficulty."

We quote Dr. Jack fully on this point because he was one of the first to introduce this form of plugging instruments. We did intend following out with some care, the different orms and preparations of gold which this change in the method of filling has introduced. But this would take me far beyond the time allotted me this evening. We shall therefore only allude to this part of the subject incidentally. Much discussion and experiment has been had with reference to the adaptability of the different preparations of gold to meet the wants of the profession in enabling them to arrive at the results desired in filling teeth. Some contend that, the form of gold which will most perfectly adapt itself to the walls of the cavity and do this with the least danger of breaking the walls when frail, is the best; while others contend that, the form of gold which most perfectly welds itself into one homogeneous mass is the best. Some contend for very light and some for very heavy foil. We think that the different conditions of the cavity to be filled should very much govern us in the use of these different preparations of gold. We should all rejoice that we have so many different varieties to select from. We have besides, the different preparations of gold foil, such as the adhesive, the soft and non-adhesive, the crystal, the corrugated and many others. The sponge gold, Watt's crystal gold, Morgan's plastic, Leslie's crystalline, Lamm's shredded gold and we believe many other kinds, all possessing some admirable qualities, and all our manufacturers are making laudable efforts to meet every requirement of the profession, so that if we needed gold in some form to fill teeth in a pool of saliva, they would try and make it; indeed Lamm's shredded gold claimed this property and but for the rubber dam would have been very popular.

Gentlemen have taken great pains to test the varied properties of all these different kinds of gold. Dr. Frank Abbott, in his article on light and heavy gold foil, and crystal gold, has given us the results of his experiments which are worthy of a passing notice. They are somewhat verified by the experiments of Dr. McQuillen, but Dr. Arthur claims he can produce different results. Dr. Abbott took prepared cavities in steel, like those in teeth to be filled, and then with an automatic plugger, with the same weight of stroke, filled the same cavities with adhesive and non-adhesive gold foil, and also Watt's crystal gold. He took adhesive gold foil running up from No. 2 to 240 grains, and that and non-adhesive foil from 3 to 6, and Watt's crystal gold. The time for filling each cavity and the amount of gold by weight used in each is given.

We select the shortest and longest of each. No. 2 adhesive took fourteen minutes to introduce the filling, No 240 took thirty-six minutes, No. 3 soft or non-adhesive foil took eleven and a half minutes and No. 6 took sixteen and a half minutes. Watt's crystal gold took fourteen minutes. The plug of No. 2 adhesive gold foil weighed 15½ grains and that of No. 240 weighed 15 grains. Non adhesive No. 3 weighed 17 grains and No. 6 also 17 grains. Watt's crystal gold weighed 14 grains.

To test the adaptability of the different preparations of gold he made small cuts with an engraver in the walls of the cavity and the result of the experiments he reports as follows: He says, "I found that Nos. 3, 4, 5 and 6 non-adhesive gold foil and the crystal gold plugs all presented the appearance of having been perfectly adapted to the cavities in every particular, while all the other fillings were more or less imperfect; even the very light numbers 2, 3 and 4 presented innumerable minute openings into the body of the plug, and the fine cuts made by the engraver, were not perfectly filled." And he further remarks from No. 10 to 240 there was hardly an approximation to adaptability. The cuts of the groves were not filled; every point where the gold was folded in the least, an open space was left; in short he says, they presented an appearauce which might very appropriately be called the height of imperfection. We hope the last five years may have worked out somewhat different results.

In speaking of the changes which have been wrought in the mode of filling teeth, a few remarks are specially appropriate with reference to the reintroduction of the mallet, which appears to have been effected by Dr. Atkinson. The introduction of cohesive gold and restoration of broken teeth to their original form, necessitated for the welding of the gold a more efficient force than that of hand pressure, and the mallet secured this. Used judiciously, it certainly is an admirable instrument, a great labor saving one to the operator. We doubt its potency as a remedy for periostitis or its special soporific power. Neither can we admit that because a filling has been driven in with a twelve ounce mallet, that it must be super excellent. As yet we can hardly understand why cohesive gold consolidated against the frail walls of a cavity is less apt to fracture it than soft foil packed by hand pressure. The use of this instrument as well as the use of cohesive gold is so indispensable in securing certain rusults now obtained, that they will ever be fully appreciated by the profession, without any claim that can not stand the test of strict scientific principles.

But this welding principle of gold which necessitated the use of the mallet, also demanded something more; for even these in a moist mouth or cavity, with the aid of serrated pluggers, failed to secure permanent work. Hence just at the right time we have that most admirable of all contrivances, the rubber dam introduced. It is this after all, which makes the others of real value.

For this valuable method of keeping the cavity dry while filling a tooth, we are indebted to Dr. Barnum, and also for the fact that one preparation of rubber so essential for our success in practice, comes not to us through the grip of an intolerable patent.

We believe its introduction is destined to revolutionize the use of those fine instruments for cutting up each layer of gold as introduced, and making a honey comb of all the interior of our filling. We mean the fine serrated pluggers.—Recollect gentlemen it takes time to test any mode of practice and if our filling is to be one solid mass of gold welded in a homogeneous body, its particles are not to be held together by innumerable pits made in each layer of gold, for the retention of the next one added. This interlacing may not be welding and unless truly and thoroughly welded, may not stand the test of time. These suggestions are thrown out that you may not too hastily overlook questions which are worthy of most careful observation as you now engage in practice.

Time will not permit the extended review we intended. We find so much that is valuable which has been introduced in the last thirty years, that it would take hours to even notice them. There is the work of your worthy Professor of operative dentistry which contains almost everything new and old on this subject, which we have not had time to speak of, which, however, must be familiar to you all. Indeed we have been only able to touch upon the leading points, which have marked the progress thus far made.

Gentlemen, if in thirty years you can show as great progress, you need not blush for dental science.

We feel that we can not close this part of our subject without an allusion to the most beautiful and wonder working engine, introduced by Dr. Morrison, of St. Louis, with all the improvements added thereto. Will thirty years or the next centennial give anything better? If so, you may set at ease in your operating room and let machinery do all your work.

We are now passing through what might be called a testing period. So much of that which relates to the peculiar kind and quality of gold we use and the manner of its introduction is new, that we must have time to thoroughly test all these things. It is not all gold which glitters, and time alone will sift out all the excellences which will be of real service. We know we are making progress, that we are working out results which heretofore were not obtained. We know too, that many splendid conceptions which promised great and grand results, are crumbling under the tooth of time.

These things, gentlemen, must interest you as members of an important profession, you are thus enabled to see the efforts which have been made and the results which have followed. There is no department of the healing art, requiring more patient perseverance and untiring industry, than that you have chosen. Think not that perfection has yet been obtained. We look to you for continued progress. The honor and character of the profession is in your hands, the more illustrious this is made, the more useful you may become. He who loses sight of his profession's character, must soon suffer in his own. If the profession is worthy of your choice, it should receive all your energies, without this you can not expect success.

Gentlemen graduates, you have now received your sealed credentials. Its device is an open book with this motto, "Science combats disease." You now go forth to test your weapons in this great combat. May they ever be directed by those great principles on which our science is based. Thus directed and skillfully used, success will crown your efforts. Your attention has been drawn to that unwearied labor and experimental investigation by which professional excellence has been attained. This hall with its associations, cluster around you, these will soon become a part of the past, but the present and the future, what are they? We cling to the present, the chain that binds us can scarce be severed. We linger here, but time waits not for us and the busy rattling world moves onward. The spell must be broken, for the future is in prospective and gilded hopes like airy castles, are beckoning you away. Sweet home, father, mother, sisters, brothers and loved ones, and perhaps other ties, scarce revealed, send a thrill of joy and hope to your hearts. Blest Elysian, may its spell lead to happiness and love.

The future then, what is it? Will it be a realization of these fond hopes, a crowning success in the ambition for fame and position? Whether this be so or not, recollect you have in part at least the shaping of your future. Aim high the upper seats are never crowded. You leave us with the best wishes of the Faculty for your long continued usefulness and prosperity. There is not a step in your future life, but will be watched with interest by your friends as well as by the friends of science. There will be no trophy won, no success attained, no scientific improvement made, but will be hailed with pleasure, and each wreath of fame thus acquired; will be an additional gem, in that crown which shall adorn your *Alma Mater*. In behalf then of the Faculty, we welcome you into the profession you have chosen. It is already adorned by names which dazzle on the page of history, they are bright stars in the world of science; their light is shed on every department of our art, their researches have opened up a vast field for your exploration. May your pathway be illumined by the truths they have unfolded and each step you take be made more and more clear by your labors, until this parting farewell shall be lost in the brightness of the future.

